# Neonatology in Austria: ethics to improve practice

**DOI:** 10.1007/s11019-020-09943-6

**Published:** 2020-03-07

**Authors:** Michal Stanak

**Affiliations:** 1 Austrian Institute for Health Technology Assessment, Garnisongasse 7/20, Vienna, 1090 Austria; 2 Faculty of Philosophy and Education, University of Vienna, Vienna, Austria

**Keywords:** Neonatology, Limit of viability, Ethics, Virtue ethics

## Abstract

In the world of Austrian neonatal intensive care units, the role of ethics is recognized only partially. The normatively tense cases that are at the backdrop of this essay concern the situations around the limit of viability (weeks 22 + 0 days to 25 + 6 days of gestation), which is the point in the development of an extremely preterm infant at which there are chances of extra-uterine survival. This essay first outlines the key explicit ethical challenges that are mainly concerned with notions of uncertainty and best interest. Then, it attempts to elucidate the less explicit ethical challenges related to the notion of nudging in the neonatal practice and argue that the role of ethics needs to be recognized more—with the focus on the role of virtue ethics—in order to improve the practice of neonatal medicine.

## Introduction

In the world of Austrian neonatal intensive care units (NICUs), the role of ethics is recognized only partially. The designated place for ethics is mainly outsourced to ethics committees that sometimes include the treating NICU professionals and sometimes consist of independent experts and heads of neonatal units (Stanak and Hawlik 2017). Depending on the hospital, these committees are established either formally or informally and their function is to support the decision-making process especially in the normatively tense cases (Stanak and Hawlik 2017). It is rarely so that a form of ethics moderation supporting the NICU team as such is present (Stanak and Hawlik 2017). The normatively tense cases that are at the backdrop of this essay concern the situations around the *limit of viability* (weeks 22 + 0 days to 25 + 6 days of gestation), which is the point in the development of an extremely preterm (EP) infant at which there are chances of extra-uterine survival (Ehrenkranz and Mercurio 2017). Currently, according to the Austrian guideline on the management of EP infants, comfort (palliative) care is recommended in 22nd week, shared decision-making with parents in the *grey zone* in 23rd week, and active care interventions from 24th week onwards (Berger 2017). This essay first outlines the key explicit ethical challenges present at the limit of viability. Then, it attempts to elucidate the more subtle (less explicit) ethical challenges related to the notion of nudging in the neonatal practice and argue that the role of ethics needs to be recognized more—with the focus on the role of virtue ethics—in order to improve the practice of neonatal medicine.

## Explicit moral challenges in the neonatal practice

### Uncertainty of data

The moral challenges discussed in the neonatal literature take their shape and form at the backdrop of their specific social, cultural, religious, and legal contexts. For the most part, however, the key challenges stem from the uncertainty of data and the ambiguity of the notion of best interest. Because the probability of healthy survival increases along with the weeks of gestation (Myrhaug et al. 2017), precision of the baseline data indicating the infant’s stage of development is of the utmost importance. The exact data, however, is covered with a veil of uncertainty that is represented by a probability range. The baseline data measurement of the infant’s stage of development can vary with the assessment tool used: while crown to rump measurements can vary ± three days, the New Ballard Exam by as much as two weeks (Leuthner 2014).

The same lack of certainty plays its role also in the assessment of outcome data. Different countries and different hospitals within countries produce different outcomes (Hawlik and Stanak 2017). The differences in approach seem to be connected to expertise (Hawlik and Stanak 2017), regionalization of care (Marlow et al. 2014), resource capacities as well as to values and norms of particular societies (Lantos and Meadow 2009). For instance, when a policy limits “treatment for infants born at 24 weeks of gestation”, it will inevitably lead to “low survival rates for those infants. The low survival rates will seem to justify and validate the policy, even if the true causal relationship runs in the other direction” (Lantos and Meadow 2009).

Uncertainties about baseline and outcome data further couple with the empirical uncertainty of what it is like to live through the experience of comfort care, active treatment that leads to death, or active treatment that leads to neurodevelopmental impairment (NDI) (Dupont-Thibodeau et al. 2014). That, in turn, leads to further ethical uncertainty. The lack of clarity in these three categories of data (baseline, outcome, and empirical) invites value judgments to be made by the NICU professionals (Leuthner 2014).

### Best interest

Another key moral challenge stems from the ambiguity about the best interest of the EP infant (Stanak 2018). Because deciding in the *grey zone* of gestation is a matter of surrogate decision-making the rightness or wrongness of which cannot be verified with the EP infant until considerably later, the question of surrogate consent and the connected question of best interest are at stake. The challenges are that it is unclear whose best interest is to be taken into account (family vs infant) and what that EP infant’s best interest looks like amidst the prospects of low quality of life (QoL) and long-term NDI (Leuthner 2014).

Furthermore, discerning what exactly constitutes the best interest of an EP infant can be a source of conflict between NICU professionals and parents, as well as within NICU teams. The judgment on best interest rests on the individual judgment of whoever evaluates the case (Larcher 2013). The problem may arise, for instance, when looking at quantitative and qualitative futility of active care. Quantitative futility entails that an intervention does not work and qualitative futility entails that an intervention is not worth it (Dupont-Thibodeau et al. 2014). At times, however, NICU professionals may conflate these two meanings and communicate to parents their judgment on qualitative as opposed to quantitative futility—thus making a value judgment on what the threshold QoL worth striving for is.

The above outlined moral challenges connected to uncertainty of data and the notion of best interest are much discussed in the literature. More implicit challenges that are given much less attention to are discussed below. They revolve around the notion of nudging in the decision-making process surrounding the limit of viability and the related notions of paternalism, freedom of choice, and coercion.

## Implicit moral challenges in the neonatal practice

### Paternalism and choice architecture

In the decision-making around the limit of viability in Austria, shared procedures have been long established in the morally ambiguous *grey zone* (now, the 23rd week of gestation (Berger 2017)) where it is ultimately the parents who act as surrogate decision-makers on behalf of the EP infant. It is the so called *zone of parental discretion* (Gillam et al. 2017). As parents participate in arriving at the decision, paternalism in its traditional negative meaning associated with coercion is seemingly not an imminent matter of concern. However, particularly because of the challenge of surrogate consent, I want to suggest that there is a need to consider a more implicit (and thus potentially also coercive) aspect of paternalism, namely that of nudging in doctor patient communication that may occur due to the presence of professional as well as parental biases in the decision-making process (Lantos 2018).

### Default bias example

With respect to behavioural psychology, there is a spectrum of cognitive biases present in any case of decision-making—including the decision-making at the limit of viability. For instance, the option presented as default seems to be the one that people tend to follow as deciding against the default often involves extra effort termed as *friction cost* or *hassle factor* (Halpern 2015). In case of NICUs, hypothetical case scenarios confirm the same trend. When lay volunteers were randomized to receive either resuscitation or comfort care as the default option for an EP infant born at 23^rd^ week of gestation, participants had the significant tendency to follow the default (Howard et al. 2012).

In the communication between NICU professionals and parents of EP infants, the impact of *default bias* has the potential to anchor the parents on either following the active or comfort care option. The challenge is that it is very hard to communicate to parents without revealing what the institutional default is. That is because every NICU has its own institutional statistics on when active care is worthwhile that, in case of very high income countries, vary between weeks 22 and 25 of gestation (Guillén, et al. 2015). These statistics themselves reveal differences between institutions and between countries, which account to institutional biases on their own. Hence, communicating to parents that at week 24 of gestation, the respective NICU goes for active care by default may automatically set-up the *default bias*.

The list of biases, however, does not end here. Affective as well as cognitive biases play their role on the side of parents as well as NICU professionals. For instance, *affective forecasting* captures the tendency of NICU parents to predict future states of their infant inaccurately or *overconfidence bias* captures the tendency of doctors to overestimate how much they know and how reliably they know it (which is of particular importance in the NICU due to the issues with uncertainty) (Janvier et al. 2014). Even though it sounds counterintuitive that nudging might be going on in the *zone of parental discretion*, the process of NICU decision-making is loaded with a spectrum of biases that make nudging (in part) inevitable. I outlined a list of more or less avoidable biases in NICU decision-making here (Stanak 2019). The *default bias* (as well as other biases the sample of which I discuss later on in this essay such as *framing effect* or *bandwagon effect*) are potentially present in the decision-making around the limit of viability and the intentional work with these biases is what it means to nudge (Stanak 2019).

The two key moral challenges with nudging are going to be examined below. The locus of the first one concerns agency of the nudger, while locus of the second concerns the challenge of coercion of the nudged. Also, due to the ongoing confusion regarding the use of the term nudging, the key distinctions will be made explicit.

## Analysis of nudging in NICUs: definitions and moral challenges

### Definition of nudging and choice architecture

It is necessary to clarify the terms used in this essay and distinguish between choice architecture and nudging. As Sunstein and Thaler define it, nudging is “any aspect of the choice architecture that alters people’s behaviour in a predictable way without forbidding any options or significantly changing their economic incentives” (Thaler and Sunstein 2008). What this distinction means in the situation of shared decision-making with parents in the NICU is the following. In conversations with NICU professionals, parents get a particular set of choices laid out in front of them. On one level, parents choose between active and comfort care. On another level, these two options can be presented in many different ways: with the use of a default, with a positive or a negative frame, with the use of peer-pressure or without, etc. Choice architecture refers not only to *what* is being presented, but also to *how* that thing is presented (Thaler and Sunstein 2008). I use the term choice architecture to depict the state of things in which nobody wilfully interacts with the way the choice setting is architected. Nudging, to the contrary, refers to the wilful interaction with the setting—the *architecting* of *what* choices are presented and *how* they are presented.

### Definition of agency

When talking about the “wilful interaction with the choice setting”, it is important to distinguish between different dimensions of will, or agency, that are at play in nudging—particularly the psychological and the ethical (Hyman 2015). The two dimensions of agency that are in use here are *intention* and *voluntariness*.

On the one hand, intention, as a psychological concept, refers to the expression of purpose or desire (Hyman 2015). Nudging requires intentional use of choice architecture where the decision-makers use the tool for a particular purpose. For instance, the intentional use of the *framing effect* in presenting the options in a positive or a negative frame (in the frame of survival vs mortality) aims to make the parents decide in one way or another. This is supported by a randomized survey that found a trend toward a *framing effect* on the treatment preference. Participants for whom the prognosis was framed as survival and non-disability rates were more likely to choose resuscitation than participants for whom prognosis was framed as mortality and disability rates (Howard et al. 2012).

On the other hand, the dimension of voluntariness, as an ethical concept, refers to the related responsibility for nudging (Hyman 2015). Nudging requires the voluntary use of choice architecture where the decision-makers use the tool of nudging out of their own volition. An act is understood here as “voluntary if it is not due to ignorance or compulsion” (Hyman 2015; Aristotle. 2011). Hence, one ought not to bear the responsibility if compelled or forced to nudge because such an act can be categorized as done under duress (Hyman 2015). Equally, if one does not know of the fact that, for instance, negative framing of information nudges parents to choose comfort care for their EP infant (the case of ignorance), one does not bear full the responsibility for it either. Both ignorance and compulsion thus serve as exculpations.

### Moral challenges with agency of the nudger

Concerning compulsion and nudging in the context of NICUs, one needs to distinguish between two categories of situations. There are some situations when nudging can be avoided and others when it cannot. While the NICU professional can avoid using the *bandwagon effect* (by not telling parents how others decided in the specific situation), the above mentioned *framing effect* is hard to be avoided (see table in (Stanak 2019)). What is meant here is that the NICU professional simply has to communicate the message in one way or another (in a positive frame of survival or in the negative frame of morbidity) and thus nudge the parents. There is thus an extent to which nudging is considered inevitable and hence, at times, nudging parents in the decision-making process at the limit of viability may present a moral challenge for the NICU professionals as it can be seen as done under duress.

Concerning ignorance and nudging, NICU professionals are exculpated from ignoring the tool of nudging only as long as they lack the knowledge of it. They are thus to be held responsible once they know of the tool, but do not use it intentionally. What adds complexity to this debate is the controversial nature of what epistemic conditions on responsibility are (Levy 2018). While some argue that agents can be held responsible only for what they actually know (Peels 2016), others suggest that agents can be responsible for ignorant choices as long as they could have been reasonably expected to know the required facts about their choice (Smith 1983). Along the lines of the latter, it is suggested here that as long as NICU professionals are aware that nudging may play a role in the communication with parents of EP infants, the only options that they are left with are either to nudge or to close their eyes and hope for the best—yet bear the responsibility nonetheless (Stewart 2005). Another aspect of the moral challenge with respect to agency thus concerns the question of ignorance of the tool of nudging and the related question of responsibility.

It needs to be stressed that if no intention is in place, we do not talk about nudging, but merely about unguided choice architecture. The moment, however, that NICU professionals get to be aware of the tool of nudging, they are presented with a moral challenge of having to decide whether or not to use it. Also, nudging can happen voluntarily as well as against the will of the nudger. If NICU professionals cannot avoid nudging and thus they do not nudge (strictly speaking) out of their own volition, compulsion serves as an exculpation.

### Moral challenges with coercion of the nudged

Another set of moral challenges with respect to nudging in NICUs concerns paternalism and the related notion of coercion. The key proponents of nudging, Sunstein and Thaler, place nudging into the category of libertarian paternalism (Thaler and Sunstein 2008). While the traditional view of paternalism refers to the situation when individuals are interfered with against their will with the motivation of preventing harm or making them better-off (Dworkin 2007), libertarian paternalism aims to preserve the freedom of choice, yet still make people better-off. Sunstein and Thaler suggest that that can be done via orchestrating choices that promote the good of the agents nudged and that are in line with their own desires (Thaler and Sunstein 2008). While avoiding significant incentives and choice restriction or elimination, they claim that the sheer alteration of the choice architecture does not constitute coercion – as the agent is free to choose after all (Ploug et al. 2012). What is true then about the criticism that nudges are coercive especially when considering the neonatal clinical context?

### Coercion and agency

The first premise of libertarian paternalism that needs to be examined is whether nudges really preserve freedom of choice—while freedom is understood here as the capacity to react to reasons (Fischer and Ravizza 1998). Here, it is important to introduce a distinction between those nudges that preserve freedom in, what Saghai calls, a basic sense and those that do so in a substantive sense (Saghai 2013). Nudges clearly preserve freedom in the basic sense in that they do not foreclose options. The point of concern is, however, whether nudges preserve freedom in the substantive sense, i.e. whether they bypass an agent’s capacity for deliberation (Levy 2017). The heart of the problem is that nudges often “take advantage of non-rational features of our nature…to produce their effect” (Levy 2017). Hence, to the extent that nudges do not preserve freedom in the substantive sense, they are to be seen here as coercive.

Critics of nudging argue that by bypassing our deliberative reasoning, nudging undermines responsible agency (Levy 2017). As discussed above, the elements of agency that are particularly relevant with respect to nudging are intentionality and voluntariness. When using the tool of nudging in the NICUs, the question is whether the agents nudged can decide intentionally (or purposefully) and voluntarily (bearing the responsibility for their decision). What stands in the way of intentional and voluntary decision is the obstacle of epistemic pollution and the challenge with nudges that merely aims to change the behaviour as opposed to changing the mind.

Levy suggests that we live in an environment that is epistemically polluted (Levy 2018). What he means is that there are “agents who use mimicry and other methods as a means of inflating their pretence to expertise”, which in turn makes it hard to distinguish between a genuine expert and a charlatan (Levy 2018). This may be problematic for parents of an EP infant in the shared decision-making procedure who have to rely on information from neonatologists when making their decision about active or comfort treatment. Assuming that neonatologists are the genuine experts, epistemic pollutant googled on the internet may mislead parents in their decision-making procedure. For instance, finding an information taken out of its context about the exaggerated burden of NDIs or, to the contrary, about the hopeful chances of EP infant’s survival below the limit of viability may serve as an epistemic pollutant.

Epistemic pollution is not a source of coercion. It is merely another obstacle on the way towards deliberative reasoning and decision-making that is intentional and voluntary. Sources of coercion, however, are all the nudges that make use of the non-rational features of our nature such as those that make use of the above mentioned *bandwagon effect* or *framing effect*. It is all those nudges that do not fall into the category of what Levy calls “nudges to reason” (Levy 2017). Nudges to reason are those nudges that “increase our responsiveness to evidence” (Levy 2017). Their aim is not to directly affect behaviour, but to “affect behaviour in ways that are mediated by beliefs. They change behaviour by changing minds”- just like rational arguments do (Levy 2017). A nudge to reason must make the agent’s mind more responsive to genuine evidence (Levy 2017).

In the case of NICUs, a nudge that could qualify as a nudge to reason would be, for instance, making use of the salience bias in decision aids used in the shared decision-making procedures. Decision aids are, for instance, visual tools with short messages and graphics that depict chances of survival, situation in the delivery room resuscitation, or the risks for neurodevelopmental disabilities (Kakkilaya et al. xxxx). The aim of decision aids is to improve health literacy and not harness bad reasoning such as in situations of communication of proportional data. The aim is to prevent situations such as when patients tend to irrationally choose a procedure where the risk of death is described as 24 out of 100, but they tend not to choose the one where the risk is described as 120 out of 1000. It is the case even though the first one presents the risk of 24%, while the second just 12%, yet the number 120 is greater than 24 (Janvier et al. 2014). Working with salience, in this context, would mean making the key data noticeable with the aim of helping the parent to decide on genuine evidence. Deciding on genuine evidence would allow parents to make an intentional and voluntary decision. Hence, altering the choice architecture with any aim other than the one that aims to nudge parent to reason is seen here as coercive. What constitutes coercion then are all the influences that aim at change in behaviour and not at change of mind. Of all the nudges, it is only those that attempt to change the mind that lead the parents to make an intentional and voluntary decision. Surely, decision aids also need to be subject to quality control as they too can present the developer’s bias and steer the parental decision towards active or comfort care (Guillen and Kirpalani 2018).

### Coercion and agent’s good

The second premise of libertarian paternalism is that preserving the freedom of choice, it still makes people better-off. In case of NICUs, to know this would require the nudger to know what the best interest of the EP infant and the family is. However, not only are the clinical facts about what exactly is beneficial in part unknowable, but also the meaning of the word *best* is inevitably connected to the subject who evaluates the case. Different subjects, different stakeholders, may interpret the best interest of the EP infant differently.

This can be illustrated on the case of cochlear implants for children. At the beginning, when the implants were introduced to the clinical practice, the clinical staff valued the intervention differently from the way parents did. While the clinicians praised the fact that the use of cochlear implants brought about partial hearing, the parents objected that the technology represented a negative value judgment on deaf culture and upon its most important feature, sign language (Daniels and Wilt 2016). In case of NICUs, the evaluation of both best interest as well as QoL of the infant and the family is subject to individual judgment (Larcher 2013). Opinions on the threshold of QoL may differ and just as the parents valued the cochlear implant intervention differently to the way clinicians did, it may also be the case when passing judgments on the best interest of EP infants at NICUs. For that reason, nudgers cannot know what, in fact, makes the agents nudged better-off and hence all other nudging in NICUs than nudging to reason constitutes coercion.

As Mill puts it “a man’s mode of laying out his existence is the best, not because it is the best in itself, but because it is his own mode” (Mill 2011). Scrutinized as this Mill’s statement may be, it contributes well to our discussion. In the process of finding out their (and their infant’s) own best interest, parents should be nudged to deliberate (via nudges to reason) rather than nudged to act in ways the NICU professional perceives as best. This is particularly at stake in situations when the parents are uncertain about their decision (and so they do not know themselves what contributes to their and the infant’s best interest). In these situations, the promise of making the nudged better-off gets to be particularly ambiguous. In these vulnerable situations when the subtle use of nudging may steer the parental decision, nudging to change behaviour constitutes coercion.

To sum up, even if the choice is *just* guided by choice alteration, in the context of neonatology, all the nudging that aims merely at change in behaviour remains coercive. Even the libertarian form of paternalism interferes with the parents’ intention and voluntariness as well as their understanding of the good.

### A way forward

Taken into account all the above, the current situation appears to be particularly problematic. On the one hand, there seem to be situations when nudges are inevitable, while on the other hand, making use of the tool of nudging seems to constitute coercive paternalism. Nudges seem not to deliver upon the libertarian promise of being freedom preserving while making the nudged better-off. And, as they tend to fail in preserving freedom in a substantive sense, except for nudges to reason, the NICU professionals are, so to speak, stuck between the rock and a hard place. In other words, NICU professionals must, at time, be inevitably coercive. They may want ignore the tool of nudging altogether, but thanks to epistemic conditions on responsibility, they can be arguably held responsible as they could have been reasonably expected to know about nudging in the first place. What is then the possible way out of this? Apart from using nudging for the purpose of supporting deliberative reasoning (as outline above) (Levy 2017), I want to suggest that nudges should be dealt with transparently on the grounds of the respective professions.

Firstly, transparency should serve as a tool of quality control in order to limit the coercive threat of nudging. In case of NICUs, applying the condition of transparency would mean that in the process of antenatal counselling, NICU professionals would communicate openly about, for instance, the possible impact of the *framing effect* or the *default bias* (even though there is evidence suggesting that the impact of a default tends to persist even if the patients are aware of it (Loewenstein et al. 2015)). The agents who are nudged ought to know that they are being nudged, especially in the vulnerable context of shared decision-making procedures at the limit of viability*,* even if we run the risk of the nudge potentially losing its effect. Focusing on the meso-level of neonatal guidelines, I want to suggest that the impact of the form of communication on parental decision-making needs to be explicitly recognized on this ground. I have also argued elsewhere that this chould be done along the line of Norman Daniels’ *accountability for reasonableness* framework (Stanak 2019).

Secondly, under the above condition of transparency, I want to suggest that the tool of nudging should rest in the hands of professions that, in case of clinical medicine, use it in line with their professional commitment to serve the good of the patient. The work of NICU professionals is not only clinical in its nature, but it is also normative. Because of the above reasons of moral challenges as well as the issue of coercion and values related to paternalistic communication with parents, NICU professionals have to participate in ethics. They need to make judgments that are concerned with rightness and wrongness that stand separate to their clinical decisions. This constitutes the *moral sphere* of their profession (Stanak 2018). For instance, in communication with parents, they need to make judgments about the threshold QoL of the EP infant and hence its best interest. These are essentially value judgments and once the NICU professionals are aware of it (and hence the exculpation based on ignorance cannot be applied (Hyman 2015)), they ought to recognize the role of ethics in their profession.

I want to further argue that there is a need to recognize the role of ethics in the NICU profession more not only because of the theoretical argument above, but also because of the practical argument that recognizing the role of ethics leads to better care for infants (Stanak 2018). It is the case because of two reasons. Firstly, better care is reached indirectly via resolving the ethical tensions within teams. Resolving ethical tensions leads to better organizational culture that, as suggested by a Canadian survey, in turn has an impact on the quality of care provided (Mahl, et al. 2015). Secondly, ethics education empowers the NICU professionals when facing normatively challenging decisions. As it was suggested in a US survey, ethics education among NICU doctors played a large role in their decision-making, especially in situations when they considered active care mandatory with respect to their understanding of the notions of best interest and beneficence (Weiss et al. 2016; Weiss and Munson 2016).

Facing the explicit moral challenges connected to uncertainty of data and the ambiguous notion of best interest as well as the implicit challenges surrounding the tool of nudging, NICU professionals should recognize the normative aspect of their work. Virtues of the NICU profession are to guide the use of the tool of nudging on a case by case basis.

## Virtue ethics solution

Recognizing the role of ethics education for clinical practitioners brings the moral sphere of the profession forward. I want to suggest, however, that in order to allow NICU professionals to develop as moral agents, education itself is not enough and a more holistic virtue ethics approach has a particular role to play. Assuming MacIntyre’s position that virtues develop in practices (MacIntyre 2014), I want to scrutinize the practice, or profession in this sense (Pellegrino 2009), of neonatology to see the room for virtue ethics there. The profession of clinical medicine (and hence also the NICU professionals), are orientated toward an end (*telos*) inherent to the their profession, which is the good of the patient (Pellegrino 2009). The clinical professionals commit to act in the best interest of the patient served and through their Hippocratic oath, they enter into a covenantal trust relationship with patients. This is their *act of profession* (Pellegrino 2009). As opposed to mere occupations, professions entail a clear act of profession that is committed to the pursuit of its concrete end. On the way of this pursuit, there are specific character traits or virtues that enable one to attain the end of the profession best (Pellegrino 2009). These are the virtues internal to the respective profession.

The virtues we are concerned with are both intellectual and moral. I have argued elsewhere that intellectual virtues in the neonatal context concern the virtues of *episteme* and *phronesis* and moral virtues include the virtues of *courage, compassion, fidelity to trust*, and *integrity* (Stanak 2018). What distinguishes these moral virtues from mere character traits is their commitment to goodness (Annas 2011). Virtues are understood here as lasting character features of a person that persist through challenges and that are weakened or strengthened depending on success or failure (Annas 2011). They are reliable in the sense that they make others know what they can expect of us because the virtues are characteristic of us – they are deep features of a person. However, virtues are not merely static dispositions of character that do not change over time, but they are active features of a person that develop via selective response to circumstances (Annas 2011). Thus, they can be acquired through habituation.

Following Artistotle’s understanding of human psychology, we start with a set of dispositions and we develop them as life progresses (Aristotle. 2011). We move from an untutored state to the tutored state via formation and education (Annas 2011). The acquisition of virtues is understood to occur via habituation as it is the case in acquisition of practical skills such as playing the piano. The first step is to consciously learn the skill step by step, note by note. Through repetition of scales and simple sonatas, the pianist becomes more skilled to the point that little conscious thinking is needed to play from the notes. Observing a skilled pianist may make one think that the conscious experience present at the beginning of learning the piano has disappeared and that mere habit acquired via repetition is now present (Annas 2011). That is, however, wrong. Even though the pianist does not need the conscious input for playing, his playing is now infused with his individual expression and feeling about the piece played (Annas 2011). Hence, what makes a good pianist is not solely the mastery of his routine, but rather his capacity to put an expression into the piece played. Important to note is that just as the skill was once acquired, practice is required for the sake of maintaining its level (Annas 2011).

The same applies to virtues of the NICU profession. It has been repeatedly reported in qualitative literature that what makes the NICU professional different from the fellow obstetrician is the disposition to be *compassionate* (Tucker Edmonds et al. 2014; 2015a, b). Having compassion as a starting point, the NICU professionals face situations that challenge, for instance, their virtue of *courage* that may at times be required from them, for instance, when having to override the parental decision to withhold active care when medically indicated (Stanak 2018). Furthermore, the virtue of *fidelity to trust* and *integrity* need to be acquired by NICU professionals in practice when facing the parents of EP infants and when communicating uneasy messages to them (Stanak 2018).

Virtues require appropriate response to unique situations – especially to unforeseen situations that require prompt action – and those occur frequently in a NICU. A rational moral calculus may be the method of choice when time is plentiful, but under time constraints, it is the dispositions of character acquired through the repetitive work in the neonatal department—just like the practical skill of playing the piano—that make a good NICU professional stand out. The professional’s virtuous character is, however, not just the result of the everyday routine in the neonatal department, but it is the acquired skill that the professional developed over time thanks to which he or she can face the challenging reality of the profession and make apt decisions. The above listed virtues require precisely this intellectual virtue of *phronesis*, practical wisdom, that is an analogue to *clinical judgment* in the moral sphere. Since virtues cannot be learned in isolation, *phronesis* is needed to help one discern what action to pursue in what situation.

In terms of the practical application of virtue ethics to medical practice, research from the Jubilee Centre for Character and Virtue on Virtuous Medical Practice maps the process of formation of medical professionals in the UK and argues for more focus on the character of medical professionals. The results from their research suggest that when solving an ethical dilemma, there is a discrepancy in approach between experienced doctors and doctors in early career stages (Arthur et al. 2015). While experienced doctors rely on their judgment and character, early career doctors rely on rules. It was argued that even though the character of doctors is often recognized as important, it is not part of the formal curriculum (as formal curriculum, for the most part, puts stress on rule-based and cognitive approaches) (Arthur et al. 2015). Moreover, as part of their qualitative interviews, the “interviewees commented on the influence of role models in their initial education and subsequent practice” emphasizing the presence of a “hidden” curriculum that shapes the doctors’ early professional identity in the chosen specialties (Arthur et al. 2015).

Applying these findings to the present case, I want to suggest a stronger focus on the formation of early career NICU professionals that aims not only on the shaping of their clinical, but also on their moral judgment. Role modelling and workplace culture can thus influence the growth of NICU professionals as moral agents. As suggested by the Jubilee Centre report, “more attention should be given to training in moral character and senior staff should create more opportunities for reflecting on ethics in the workplace” (Arthur et al. 2015). It is the *phronetic* judgment of the NICU professionals that is believed to be able to help them use the tool of nudging in an appropriate way also in the conversations surrounding the limit of viability. For the flourishing of NICU professionals (or for their character development), the respective organizational culture needs to recognize the importance of their role also as moral agents (Stanak 2018). Some of the methods applied already are support from ethics committees, in-house supervision mechanisms, and ethics moderation of team discussion (that I outlined here (Stanak 2018)). The organizational culture hence needs to recognize the role of ethics in the NICU profession and support the NICU professionals on the way.

## Conclusion

I have argued in the essay that there are normative challenges at the limit of viability that force NICU professionals to participate in ethics that include the explicit moral challenges connected to uncertainty, best interest, and implicit challenges connected to coercion in nudging. I suggested that on the one hand, nudging cannot seem to escape its criticism of coercion because even if the choice is just guided by choice alteration, nudging may interfere with the agent’s intention and voluntariness as well as understanding of the good. On the other hand, however, I argued that there are situations when nudging is inevitable and hence when the NICU professional should use the tool of nudging, but deal with it as transparently as possible in line with his or her professional virtues and professional commitment. In sum, I have argued that the role of ethics needs to be recognized more and that the virtue ethics approach has a particular role to play in guiding the tool of nudging and in allowing the NICU professionals to grow as moral agents. It may have an impact on improving the practice of neonatology and hence the quality of care delivered.
